# Supplementary material for: A Precision Medicine Approach to Optimize Modulator Therapy for Rare CFTR Folding Mutants
Source: J Pers Med. 2021 Jul 7;11(7):643. doi: 10.3390/jpm11070643 (PMC8307171; doi:10.3390/jpm11070643)
Supplement: Supplementary file 1 [file jpm-11-00643-s001.zip › jpm-1282972-supplementary.pdf]

Supplementary Materials

for

A precision medicine approach to optimize modulator therapy for rare CFTR folding mutants

Guido Veit*, Tony Velkov, Haijin Xu, Nathalie Vadeboncoeur, Lara Bilodeau, Elias Matouk, and Gergely L. Lukacs*

* Correspondence: guido.veit@mcgill.ca (G.V.) or gergely.lukacs@mcgill.ca (G.L.L.); Tel.: +1-514-398-6190 (G.V.) or +1-514-398-5582 (G.L.L.)

Supplementary Figures

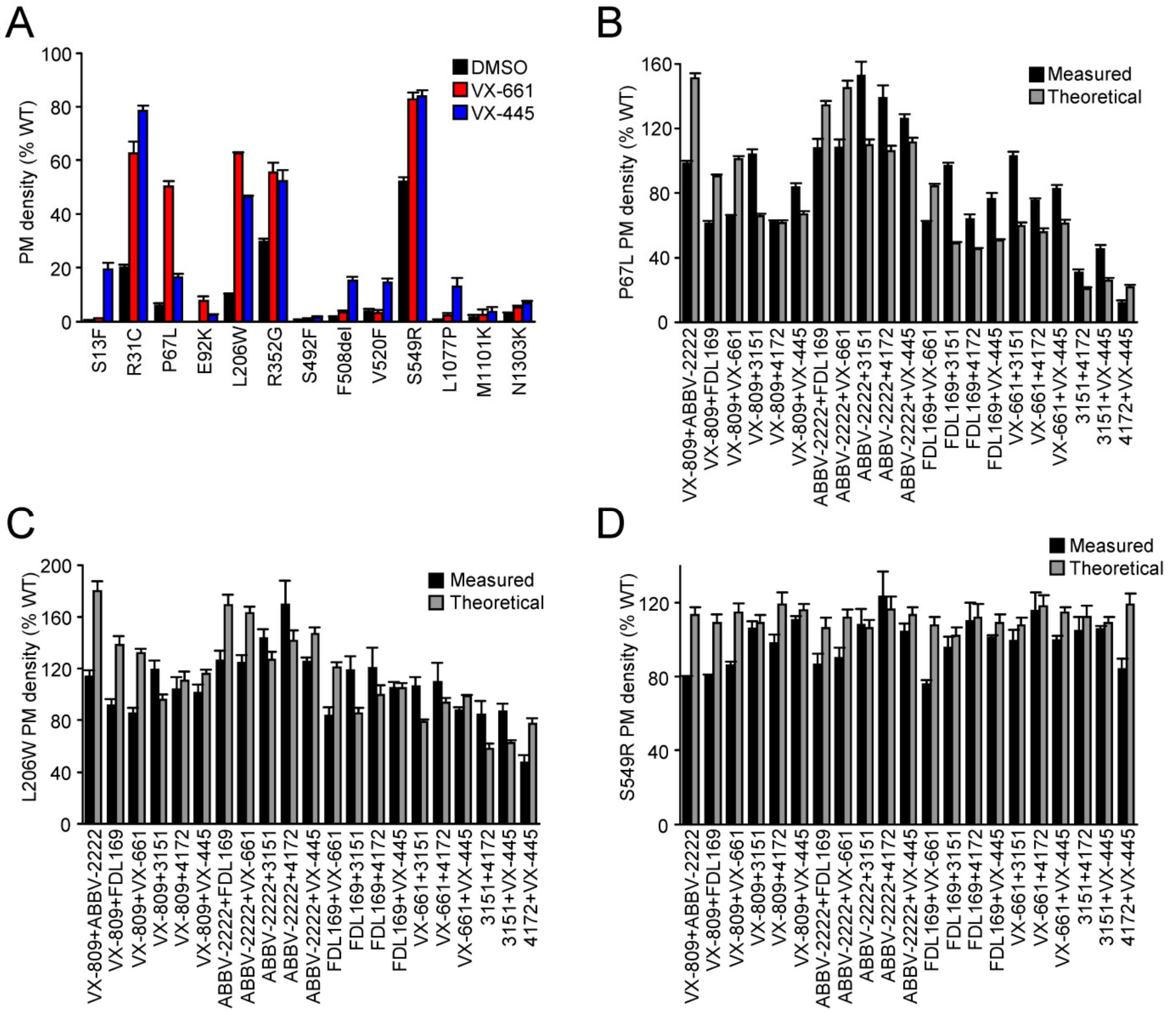

Figure S1. The effect of single correctors and dual corrector combinations on the PM density of CFTR mutants in CFBE. (**A**) PM density of the indicated mutants alone and after VX-661 (3 μM, 24 hours, 37°C) or VX-445 (2 μM, 24 hours, 37°C) treatment, expressed as the percentage of WT-CFTR in CFBE41o- cells (n = 3). The data for the S13F, R31C, E92K, R352G, S492F, F508del, S520F, L1077P, M1101K and N1303K have been published before [26] and are shown for comparison only. (**B-D**) Quantification of P67L (B), L206W (C), or S549R (D) PM density, measured by cell surface ELISA after dual corrector treatment in comparison to the calculated additivity (theoretical) of single corrector effects (n = 3). Data are means ± SEM of three independent experiments.

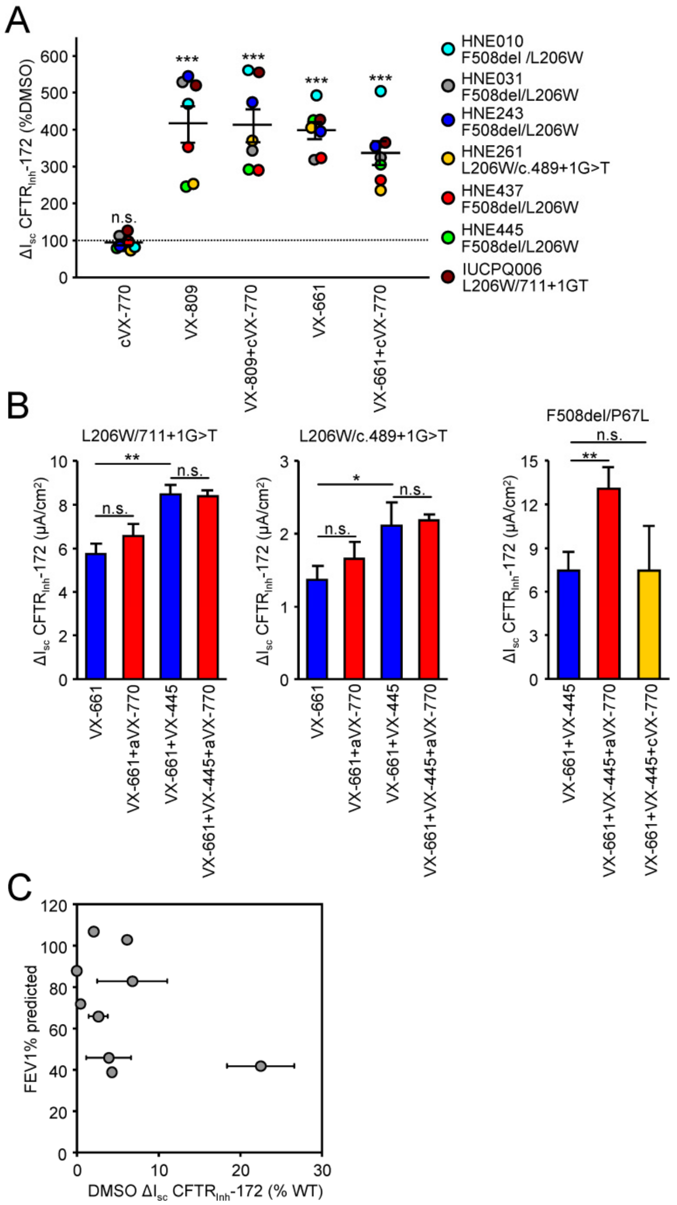

Figure S2. Functional correction of L206W-CFTR in HNE. (**A**) Effect of indicated single correctors (VX-809, VX-661 - 3μM, 24 hours) alone or in combination with acute VX-770 (aVX-770) or chronic VX-770 (cVX-770 - 1 μM, 24 hours) potentiation on the Isc of HNE from 7 patients with one L206W-CFTR allele. Quantification of the $CFTR_{Inh}$-172 inhibited current expressed as percentage of the CFTR currents in DMSO treated HNE. The same data expressed as percentage of WT-CFTR currents are shown in Figure 4B. Black lines indicate mean ± SEM. N.s.- not significant, ***$P < 0.001$ by Student's t-test. (**B**) Corrector effect in channels partially activated with 100 nM forskolin. Effect of indicated single correctors or corrector combinations (VX-809, VX-661 - 3μM; VX-445 - 2μM; 24 hours) alone or in combination with acute VX-770 (aVX-770) or chronic VX-770 (cVX-770 - 1 μM, 24 hours) potentiation on the I_{sc} of HNE from 2 patients with one L206W-CFTR allele and a splice site mutation on the second allele or on F508del/P67L HNE. CFTR-mediated currents were induced by sequential acute addition of forskolin (100 nM) and VX-770 (10 μM) followed by CFTR inhibition with $CFTR_{Inh}$-172 in an intact monolayer with basolateral-to-apical chloride gradient and the $CFTR_{Inh}$-172-inhibited currents were quantified (n = 3). Data are means ± SD of 3 measurements. N.s.- not significant, *$P < 0.05$, **$P < 0.01$ by Student's t-test. (**C**) Correlation between the basal Fsk-stimulated current (n = 3) in HNE from 9 patients with one allele of either P67L-, L206W or S549R-CFTR and the FEV1% predicted measured at the point of HNE collection.
